# A case report of delayed left ventricular rupture after mitral transcatheter edge-to-edge repair: clip entrapment in hypercontractile left ventricle

**DOI:** 10.1093/ehjcr/ytaf265

**Published:** 2025-05-26

**Authors:** Shinichi Kurashima, Makoto Amaki, Tomoyuki Fujita, Takeshi Kitai, Chisato Izumi

**Affiliations:** Department of Heart Failure and Transplantation, National Cerebral and Cardiovascular Center, 6-1 Kishibe-shinmachi, Suita, Osaka 564-8565, Japan; Department of Heart Failure and Transplantation, National Cerebral and Cardiovascular Center, 6-1 Kishibe-shinmachi, Suita, Osaka 564-8565, Japan; Department of Cardiovascular Surgery, National Cerebral and Cardiovascular Center, 6-1 Kishibe-shinmachi, Suita, Osaka 564-8565, Japan; Department of Cardiovascular Surgery, Tokyo Medical and Dental University, 1-5-45 Yushima, Bunkyo-ku, Tokyo 113-8510, Japan; Department of Heart Failure and Transplantation, National Cerebral and Cardiovascular Center, 6-1 Kishibe-shinmachi, Suita, Osaka 564-8565, Japan; Department of Heart Failure and Transplantation, National Cerebral and Cardiovascular Center, 6-1 Kishibe-shinmachi, Suita, Osaka 564-8565, Japan

**Keywords:** Mitral transcatheter edge-to-edge repair, Mitral regurgitation, Left ventricular rupture, Hypertrophied papillary muscle, Case report

## Abstract

**Background:**

Left ventricular (LV) rupture is an extremely rare but possible complication after mitral transcatheter edge-to-edge repair (M-TEER). We describe a delayed LV rupture after M-TEER that was successfully treated with surgical repair.

**Case summary:**

An 83-year-old Asian male with congestive heart failure was referred for treatment of severe mitral regurgitation (MR) due to A1/A2 segment prolapse with abnormally hypertrophied anterior papillary muscle. The patient was at high surgical risk, and M-TEER with MitraClip (Abbott Vascular, Minneapolis, MN, USA) was performed. During the procedure, an NT clip became entangled between the hypertrophied papillary muscle and the LV inferolateral wall. After disentangling the clip, we aimed the clip for a second attempt slightly towards the medial side and inserted it into the LV, avoiding interference with the subvalvular apparatus or LV wall. Grasping in this position significantly reduced MR to mild. The patient was initially stable, but sudden cardiac arrest occurred 75 min post-procedure, and subsequent echocardiography revealed massive pericardial effusion. Emergent sternotomy revealed a tear at the LV basal inferolateral wall just behind the anterior papillary muscle. Surgical patch repair and mitral valve replacement were performed, and the patient was discharged without neurological sequelae.

**Discussion:**

The entrapment of the clip between the hypertrophied papillary muscle and the hypercontractile LV wall may have caused a crack in the LV wall, disrupting the endocardium. In elderly patients with primary MR, especially those with commissural lesions and limited LV space, clinicians should be cautious of LV rupture even after the procedure.

**Learning points**

- Left ventricular (LV) rupture in mitral transcatheter edge-to-edge repair (M-TEER) is an extremely rare but possible complication.
- The clip trapped between the hypertrophied papillary muscle and the hypercontractile LV wall may have caused a crack and disrupted the endocardium of the LV.
- In M-TEER, delayed LV rupture should be recognized as a new complication, particularly in elderly patients with limited space near commissure lesions.

## Introduction

Mitral transcatheter edge-to-edge repair (M-TEER) is a minimally invasive treatment for both primary and secondary mitral regurgitation (MR) in patients at high surgical risk.^1,2^ Although M-TEER is a safe procedure, there have been fatal complications requiring open chest surgery.^2,3^ Left ventricular (LV) rupture is an extremely rare complication but has recently been reported ‘during’ M-TEER procedure.^4^ We herein report the first case of ‘delayed’ LV rupture after M-TEER successfully treated with emergent LV patch repair and mitral valve replacement (MVR).

## Summary figure

**Figure:**
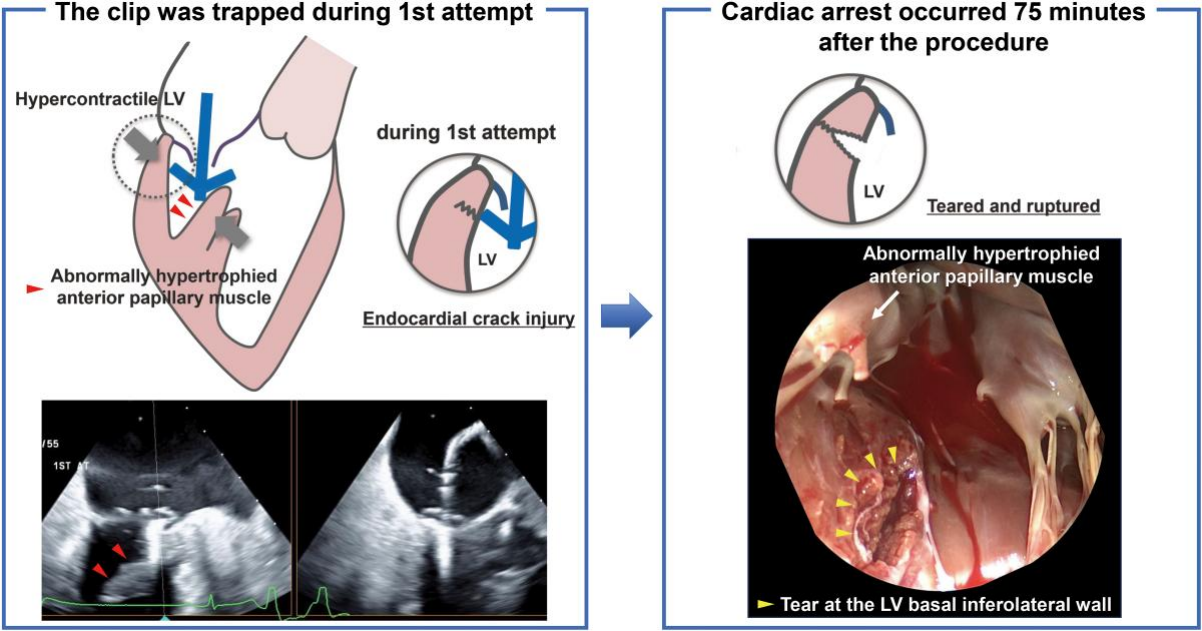


## Case presentation

**Figure 1:**
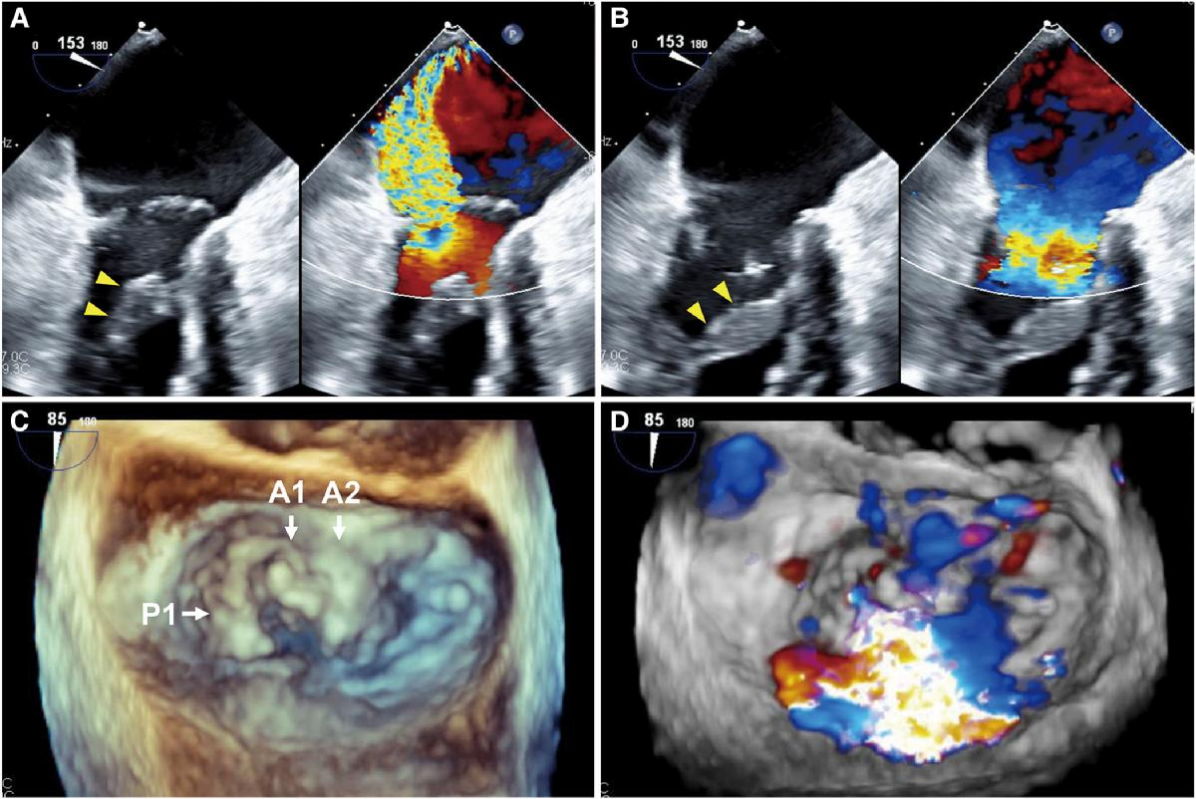
Preoperative echocardiography. (*A*) Severe mitral regurgitation due to A1/A2 segment prolapse with hypertrophied anterior papillary muscle (arrowheads) at systole. (*B*) Hypertrophied anterior papillary muscle (arrowheads) at diastole. (*C*) 3D image of the mitral valve. (*D*) 3D colour image of the mitral valve.

An 83-year-old Asian male with no prior history of cardiac disease developed exertional dyspnoea 1 month ago and was subsequently admitted to another hospital for acute heart failure. The heart failure was successfully treated with diuretics, and the patient was diagnosed with severe MR due to posterior leaflet prolapse. He had a history of diabetes mellitus, dyslipidaemia, and chronic obstructive pulmonary disease. After 4 months, he was admitted to our hospital for the treatment of severe MR. At admission, he was haemodynamically stable (blood pressure 110/68 mmHg, heart rate 81 b.p.m., and pulse oximetry 98% on room air) and in functional New York Heart Association Class II. He was taking furosemide 40 mg and tolvaptan 7.5 mg for managing fluid retention, as well as empagliflozin 10 mg and rosuvastatin 5 mg. Physical examination revealed no peripheral oedema, clear lung sounds, and an apical holosystolic murmur (Levine 3/6). Electrocardiography showed normal sinus rhythm with no signs of ischaemia or LV hypertrophy. Transthoracic echocardiography (TTE) showed severe MR, with an effective regurgitant orifice area of 40 mm² and a regurgitant volume of 68 mL, calculated using the proximal isovelocity surface area method. Left ventricular ejection fraction was preserved at 65%, with an LV end-diastolic/systolic diameter of 54/37 mm and a septum/posterior wall thickness of 9 mm. Left ventricular strain was within the normal range, and no apical sparing pattern was observed. Transoesophageal echocardiography showed A1/A2 segment prolapse with abnormally hypertrophied anterior papillary muscle (*Figure 1*; see Supplementary material online, *Video S1*), which was also observed on TTE (see Supplementary material online, *Videos S2*–*S5*).

The length of the posterior mitral leaflet (PML) was 8.7 mm, and the anterior mitral leaflet was 12.6 mm at the grasping point. The risk of surgical mitral valve repair was moderate with a EuroSCORE Ⅱ of 4.5%. Initially, we considered surgical mitral valve repair due to anatomical concerns. However, after discussions regarding his age and frailty, our heart team concluded that the patient’s surgical risk was high and M-TEER with MitraClip (Abbott Vascular, Minneapolis, MN, USA) was performed.

**Figure 2:**
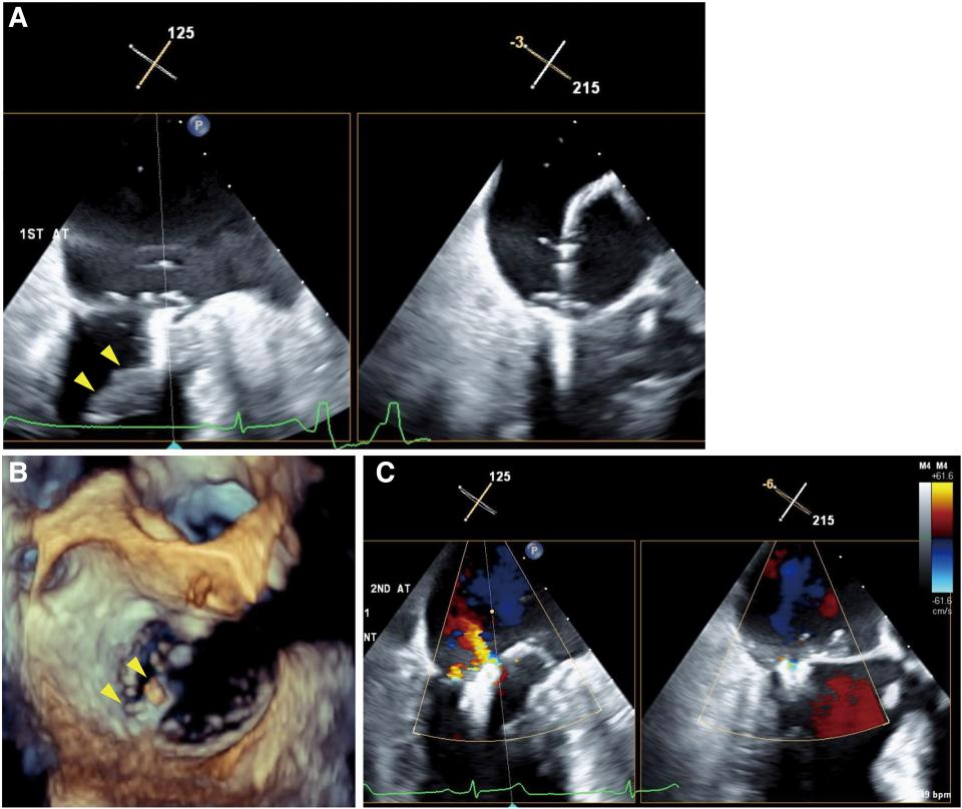
The procedure of mitral transcatheter edge-to-edge repair. (*A*) The clip was entangled to chordae and trapped between hypertrophied papillary muscle (arrowheads) and the left ventricular wall. (*B*) A 3D mitral valve en face view shows that the clip (arrowheads) was interfering with the inferolateral wall. (*C*) After the clip deployment, mitral regurgitation was reduced to mild without interference between the clip and the left ventricular wall.

**Figure 3:**
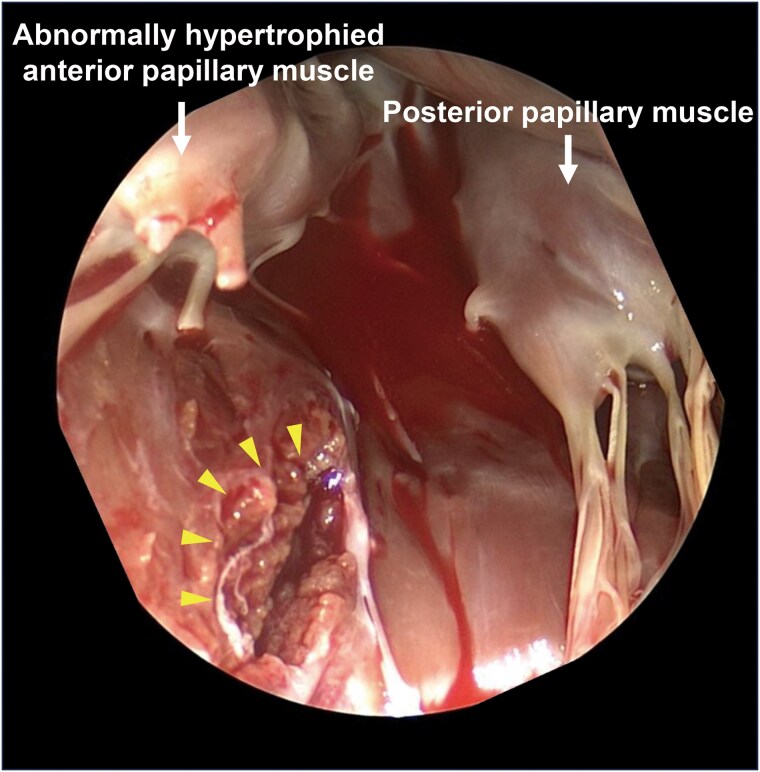
Intraoperative findings. From the surgeon's view, the tear (arrowheads) was observed in the left ventricular inferolateral wall.

Since the jet was predominantly from A1-P1 lesion and with limited space between the annulus and abnormally hypertrophied anterior papillary muscle (18.5/10.0 mm at diastole/systole), we selected an NT clip. As the clip was delivered into the A1-P1 lateral lesion, the clip was entangled in the space between the papillary muscle and LV inferolateral wall, and premature ventricular contractions appeared multiple times (*Figure 2A* and *B*; see Supplementary material online, *Videos S6* and *S7*). After releasing chordal entanglement and retrieving it back to the left atrium, we aimed the clip for a second attempt slightly towards the medial side of the A1-P1 lesion. Mitral regurgitation significantly improved to mild immediately after grasping and remained after releasing the clip without interfering with the subvalvular apparatus or LV wall (*Figure 2C*). No pericardial effusion was detected after sheath removal. At the time of extubation, the patient's consciousness was clear. His vital signs were stable, with blood pressure 92/43 mmHg and heart rate 72 b.p.m. After returning to the ward, blood pressure was slightly increased to 135/72 mmHg, but again no pericardial effusion was confirmed. However, sudden cardiac arrest occurred 75 min after the procedure. Chest compressions were immediately initiated, followed by administration of 1 mg intravenous epinephrine twice (total 2 mg) and endotracheal intubation. At the same time, TTE showed massive pericardial effusion. The patient was immediately transferred to the operating room, and extracorporeal circulation was established 15 min after sudden cardiac arrest via femoral arterial and venous cannulation. An emergent sternotomy was done and revealed a tear at the LV basal inferolateral wall just behind the anterior papillary muscle (*Figure 3*). Surgical patch repair and MVR were performed. After two more open thoracotomies (mediastinal haematoma removal and left atrial thrombectomy), the patient was discharged home without neurological sequelae. Six months after discharge, the patient was admitted with worsening heart failure. Transthoracic echocardiography at readmission showed preserved prosthetic mitral valve function with no residual MR. His condition improved with diuretics, and he remained stable during the 1.5-year follow-up.

## Discussion

This is the first report of delayed LV rupture after M-TEER. Recently, Yoshida *et al*.^4^ reported LV rupture during M-TEER. In their case, the echocardiography showed severe MR due to P3 prolapse. It was considered that the tip of the clip sank into the myocardium during the closing process due to short PML. In addition, LV rupture occurred in the acute phase immediately after grasping. In the present case, P1 length was long enough that the clip arm had not reached the annulus during closure. Moreover, from the surgical findings, the ruptured point was more lateral to the grasping point (where the first attempt was done without grasping), suggesting that rupture was not due to clip grasping of the annulus with a short posterior leaflet.

**Figure 4:**
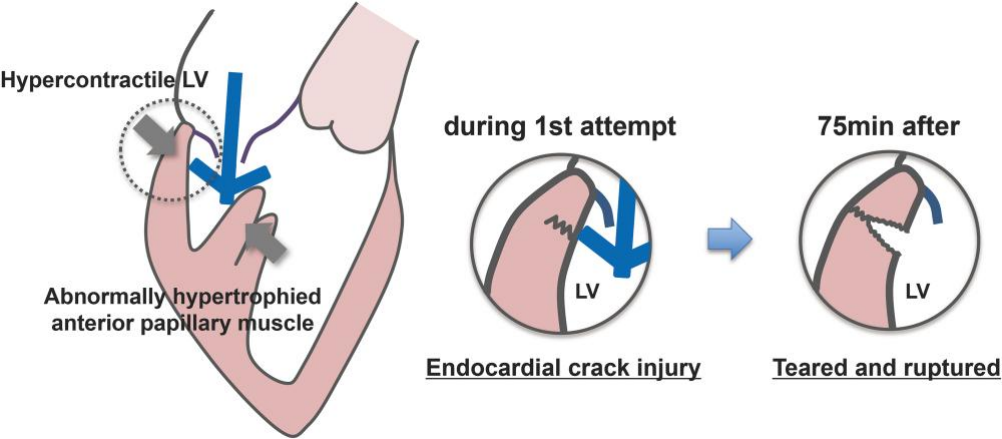
The mechanism of delayed left ventricular rupture. The open clip trapped between the papillary muscle and the hypercontractile left ventricular wall may have caused a crack in the left ventricular wall, resulting in delayed left ventricular rupture. LV, left ventricle.

The most characteristic structure in the present case is abnormally hypertrophied papillary muscle. A workup was performed for underlying cardiac disease including hypertrophic cardiomyopathy and infiltrative cardiomyopathies, especially cardiac amyloidosis. In this case, there were no findings suggestive of such conditions. During the first attempt, although we did not grasp the leaflet, the clip was trapped and sandwiched between the hypertrophied papillary muscle and the hypercontractile LV wall. At this moment, the tip of the clip arm pushed by papillary muscle may have injured and caused a crack at the LV posterior wall and disrupted the endocardium (*Figure 4*). Also, premature ventricular contractions stimulated by the contact of the clip may have worsened this mechanism by further narrowing the space in the left ventricle. Left ventricular rupture has been reported to occur in 0.8% of cases after MVR, and the mortality rate exceeds 80% when it occurs. Left ventricular rupture commonly occurs in the posterior annulus due to its lack of support from the fibrous skeleton, with advanced age being an additional risk factor.^5^ This anatomic vulnerability of the posterior annulus and tissue fragility due to advanced age may have contributed to the LV rupture in the case of Yoshida *et al*. as well as in our case. In addition, increased blood pressure after the procedure may have also played the role of crack to tear and resulted in delayed LV rupture.

Although surgery is a gold standard treatment for symptomatic severe primary MR, about half of the patients do not undergo surgery due to high surgical risk,^6,7^ and they will be candidates for M-TEER. Mitral transcatheter edge-to-edge repair has been applied even in patients with complex mitral anatomies lately. Appropriate clip selection is essential for procedural success. In commissural lesion cases, the NT series is recommended over the XT series due to high risk of clip entanglement or chordal damage.^8^ In our case, the NTW could be considered due to wide flail lesion. However, we selected NT clip with its safeness to dive into dense chordae and hypertrophied papillary muscle. While M-TEER is often favoured for its safety and durability, transcatheter mitral valve implantation (TMVI) may be a viable option in cases with complex lesions, such as multiple jet or severe calcification, which makes repair challenging.^9^ Therefore, a comprehensive anatomical assessment is crucial to inform both clip selection and the consideration of TMVI as a therapeutic alternative. In these challenging cases, the operator’s experience and the volume of the centre are significant factors in achieving successful outcomes.^8^ The eligibility for M-TEER should be evaluated with consideration of the operator’s expertise and the centre’s procedural volume.

## Conclusion

Delayed LV rupture should be recognized as a new complication in M-TEER for elderly primary MR patients with limited LV space, especially at near commissure lesions.

## Lead author biography


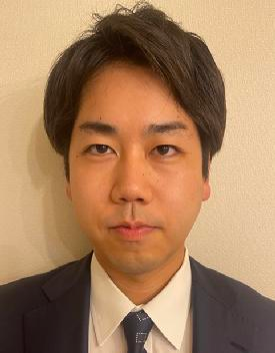


Shinichi Kurashima graduated from Yamagata University with an MD degree in 2016. He is a cardiologist at the National Cerebral and Cardiovascular Center in Osaka, Japan.

## Supplementary Material

ytaf265_Supplementary_Data

## Data Availability

The data underlying this article are available in the article and in its online Supplementary material.
